# Isolation and Detection of Exosomal Mir210 Using Carbon Nanomaterial-Coated Magnetic Beads

**DOI:** 10.3390/jfb14090441

**Published:** 2023-08-25

**Authors:** Raja Chinnappan, Qasem Ramadan, Mohammed Zourob

**Affiliations:** Department of Chemistry, Alfaisal University, Al Zahrawi Street, Al Maather, Al Takhassusi Rd, Riyadh 11533, Saudi Arabia; rchinnappan@alfaisal.edu

**Keywords:** microRNA detection, biosensors, pre-concentration of exosomes, breast cancer

## Abstract

MicroRNAs (miRNAs) are short non-coding RNAs that are found in various cellular compartments and play an important role in regulating gene expression. Extracellular miRNAs, such as those found within extracellular vesicles such as exosomes are involved in cell-to-cell communication. The intercellular transfer of miRNAs has been implicated in various diseases’ pathogenesis including cancer and has been studied extensively as potential cancer biomarkers. However, the extraction of miRNA from exosomes is still a challenging task. The current nucleic acid extraction assays are expensive and labor-intensive. In this study, we demonstrated a microfluidic device for aptamer-based magnetic separation of the exosomes and subsequent detection of the miRNA using a fluorescence switching assay, which was enabled by carbon nanomaterials coated on magnetic beads. In the OFF state, the fluorophore-labelled cDNA is quenched using carbon nanomaterials. However, when the target miRNA210 is introduced, the cDNA detaches from the bead’s surface, which leads to an increase in the fluorescence intensity (ON state). This increment was found to be proportional to miRNA concentration within the dynamic range of 0–100 nM with a detection limit of 5 pM. The assay was validated with spiked miRNA using the standard RT-PCR method. No notable cross-reactivity with other closely related miRNAs was observed. The developed method can be utilized for the minimally invasive detection of cancer biomarkers.

## 1. Introduction

Cancer is a leading cause of death globally, and the early diagnosis of the disease is of paramount importance for increasing survival rates and improving treatment outcomes [1]. Tumour biopsy is one of the most common techniques used for cancer diagnosis [2]. However, this method is highly invasive. Therefore, finding alternative diagnostic methods is highly desirable. Recently, exosomes have been identified as potential biomarkers for a variety of diseases, including cancers [3,4]. Exosomes are found in various body fluids including blood, urine, and saliva, and are made up of lipid bilayer cellular vesicles with a maximum size of 150 nm [4]. They are secreted by all types of cells and contain many important biomolecules, such as DNA, RNA, microRNA, and proteins. In addition, they are transported from the donor to the recipient cells serving as an intercellular communication system [5], thus providing a wealth of information when utilized. Exosomes play a major role in promoting tumor metastasis by carrying the growth-promoting genes to initiate the proliferation of cancer cells. For example, it was reported that cancer cells exchange exosomes in lung tissue, which was observed using green fluorescent protein (GFP)-tagged CD63 probe [6]. Also, it is believed that exosomal miRNA can mediate and silence the downstream genes and trigger tumorigeneses in nontumorigenic epithelial cells [7].

MiRNAs are small non-coding molecules with 18–22 nucleotides in length found in most living organisms that are involved in the post-transcriptional regulation of gene expression [8]. MiRNAs are found in various body fluids and secreted outside the cells through extracellular vesicles: the exosomes. The altered expression of miRNAs is also associated with many cancer progressions, including breast, lung, and prostate cancers [9], thus illustrating a high potential as clinical biomarkers for the non-invasive diagnosis and treatment of tumours. MiRNAs can be extracted from the exosomes and consequently utilized in different biological assays [10,11]. Due to their stability within the exosomes, the utility of these molecules has attracted significant attention as biomarkers for the early and non-invasive diagnosis of various cancers [12,13,14].

Exosome isolation is one of the most challenging tasks due to its small size, low abundance, and existence in a heterogeneous population of vesicles. A variety of exosome isolation methods have been exploited, including ultrafiltration, ultracentrifugation, density gradient separation, size-exclusion chromatography, polymer precipitation, and immunoaffinity [15,16], and several isolation kits are commercially available [17]. The microfluidic-based separation technique allows the extraction of biological entities from a tiny sample volume by exploiting various separation principles using intrinsic forces (e.g., fluid dynamics) or extrinsic forces (e.g., magnetic and electric fields) and different properties of the analyte. The integration of microfluidic and magnetic separation into exosome sample preparation offers several advantages, including high purity, high throughput, and low sample volume. Currently, exosome extraction methods employ antibodies as recognition elements. However, this method does not enable the non-destructive release of exosomes, which may affect the biofunction of the natural exosome and lead to false results. Aptamers are chemically synthesized oligonucleotides, which are selected by Systematic Evolution of Ligands by exponential enrichment (SELEX) processes and can be used as an alternative to antibodies (chemical antibodies) for the isolation and separation of exosomes [18]. The SELEX process involves multiple rounds of selection and amplification of specific nucleic acid from a large pool of random nucleic acid sequences. The sequences that bind to the target molecule are separated from the non-binding sequences and amplified via polymerase chain reaction (PCR). Aptamers can be selected to bind with high specificity to a wide range of target molecules, including proteins, small molecules, and cells. This allows for more precise targeting than antibodies, which can sometimes cross-react with other molecules. When binding to a target, the single-stranded DNA or RNA of the aptamers forms a unique 3D structure under ideal physiological conditions [19].

**Figure 1:**
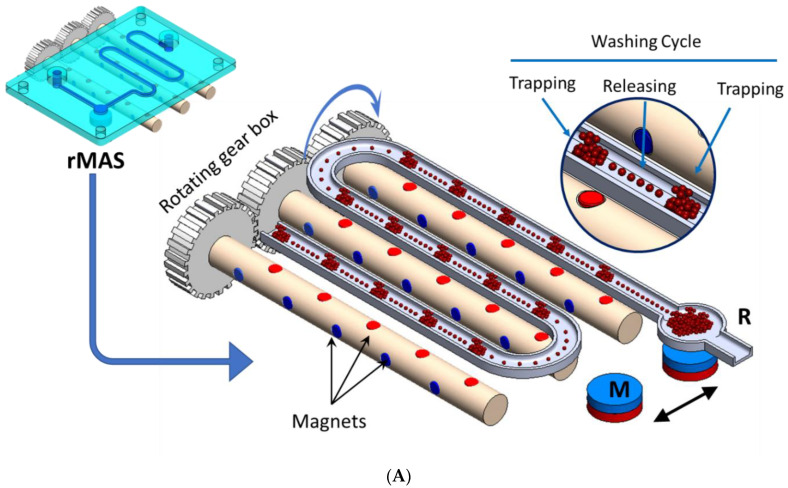

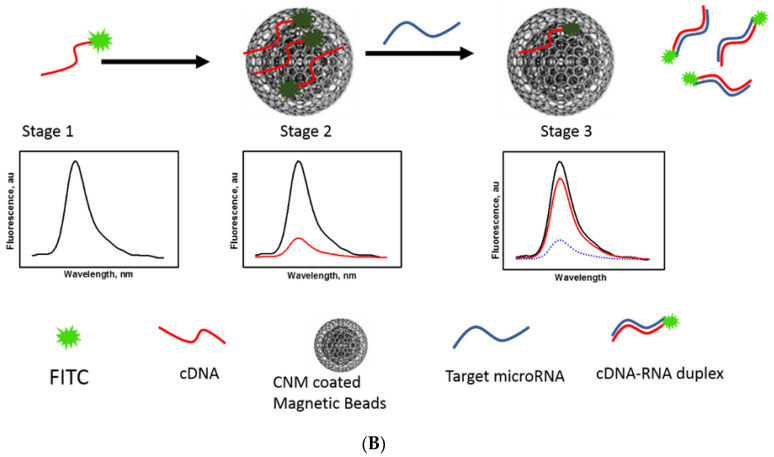
(**A**) Schematic drawing of the magnetic separator showing the rotating magnetic system under the microfluidic chip in detail. A zoomed view of the beads “trapping and releasing” is shown in the inset. M is the permanent magnet used to trap the beads after purification and R is the collecting reservoir. (**B**) Schematic of the fluorescence switching mechanism in the CNM-coated magnetic nanobeads fluorescence assay for the detection of mir210. Stage 1: The FAM-labeled cDNA of mir210 has a high fluorescence signal. Stage 2: In the presence of CNM-coated magnetic beads, the fluorescence is quenched. Stage 3: Upon introducing mir210, the cDNA detached from the CNM surface and duplexed with mir210, which led to an increase in the fluorescence intensity.

In this study, aptamer-conjugated magnetic nanobeads were employed in a microfluidic-based magnetic separation system for the isolation of exosomes from cell culture supernatants via trapping and releasing processes (Figure 1B). The exosome particle size was determined using the dynamic light scattering method. Due to the physical adsorption on the surface of the CNM, the fluorescence of the FAM-cDNA was significantly quenched (OFF state), as illustrated in Figure 1A. But in the presence of Mir210, the cDNA detached from the surface and fluorescence increased significantly (ON state). The limit of detection of Mir210 was estimated from the calibration curve. The detection method was compared with the standard RT-qPCR method.

## 2. Materials and Methods

### 2.1. Chemicals

Carbon-coated magnetic (CCM) beads were purchased from Turbo beads (Zurich, Switzerland). Information about the synthesis and chemistry of the beads can be found elsewhere [20]. Penicillin-streptomycin was purchased from Gibco Life Technologies (Carlsbad, CA, USA). Sodium chloride (NaCl), magnesium chloride (MgCl_2_), phosphate-buffered saline pH 7.4 (PBS), tris(hydroxymethyl)aminomethane (tris-base), boric acid, ethylenediaminetetraacetic acid (EDTA) disodium dihydrate, sodium azide, and hydrochloric acid were purchased from Sigma-Aldrich (St. Louis, MO, USA; https://www.sigmaaldrich.com/united-states.html (accessed on 10 February 2021)). The size exclusion chromatographic column was obtained from Izon Science Ltd. (Lyon, France). Amicon Ultra Centrifugal Filter (0.5 mL) was supplied from EMD Millipore (Sigma, Burlington, MA, USA; https://www.merckmillipore.com (accessed on 10 May 2020)). DNA purification kit (Qiagen, Hilden, Germany), RT2 first strand kit (Qiagen, cat No 330401), and purified labeled and unlabeled oligonucleotides were purchased from Metabion International (Planegg, Germany; http://www.metabion.com (accessed on 10 May 2021)), as shown in Table 1 and Table 2. Luciferase-expressing 4T1 murine breast cancer cells (4T1-Luc2) were purchased from PerkinElmer and cultured as reported previously [21].

### 2.2. Instrumentation

Concentrations of oligonucleotides and proteins were estimated via absorption at 260 nm and 280 nm using NanoDrop 2000 (Thermo Scientific, Ottawa, ON, Canada). Fluorescence signals of fluorescein-labeled cDNA and the cDNA-miRNA duplex were monitored using the Nanodrop ND3300 fluorospectrometer (Thermo Scientific, Ottawa, ON, Canada). Light-emitting diodes (LEDs) in the range of 470 ± 10 nm were used as an excitation source to excite the sample. The fluorescence intensity of the samples was observed at 515 nm. All the experiments were carried out using a binding buffer (pH, 7.4) at room temperature and repeated three times. The exosome particle size was determined by the dynamic light scattering method using the Malvern pananalytical instrument (https://www.malvernpanalytical.com (accessed on 10 September 2022)) with a monochromatic laser.

### 2.3. Exosome Isolation, Quantification, and Particle Size Determination

Luciferase-expressing 4T1 murine breast cancer cells (4T1-Luc2) were cultured in RPMI-1640 culture media containing 10% heat-treated fetal bovine serum (FBS) and 100 unit/mL penicillin-streptomycin (Gibco, Life Technologies, Carlsbad, CA, USA) at 37 °C in a humidified atmosphere containing 5% CO_2_ until 80% confluence. The cells were washed 3 times with PBS buffer and subsequently cultured in a serum-free medium for 48 h. The cell culture medium was collected and centrifuged at 500 g for 5 min in duplicate. The supernatant was concentrated using a filter with a 100 KD cut-off to a final volume of one-tenth of the original volume. The concentrated solution was then added to the Eqv column, which was pre-equilibrated with PBS buffer at room temperature, and the fractions were collected up to 1 mL. The collected fraction was concentrated using a 100 KD Amicon filter to a final volume of 150 µL. The concentration of the total protein in the extracted exosome was estimated as described elsewhere [22], and the total amount of particles was calculated from the protein content. The particle size of the extracted exosomes was measured using a Zetasizer Nano ZS equipped with a 633 nm laser (Malvern Instruments, Malvern, UK). One mL of the diluted sample was transferred to a one cm path-length cuvette. The sample was equilibrated to room temperature before size measurements.

### 2.4. Magnetic Bead-Based Isolation of Exosomes (Apta-Magnetic Separation System)

To extract the exosomes from the cell culture supernatant, a dynamic magnetic separation separator was custom-fabricated, based on the concept of flow-through “trapping and releasing” of magnetic beads in a microfluidic channel [23]. The separator employed a rotating magnet assembly, which comprised an array of small Neodymium Iron Boron (NdFeB) disc magnets, each with a diameter of 1 mm. The magnets were embedded in three cylindrical rotatable brass rods, with a diameter of 5 mm, and arranged in an alternating magnetic polar orientation, i.e., the pole direction of each magnet was perpendicular to that of the next magnet with an inter-magnet spacing of 10 mm. Cylindrical holes with a diameter of 1.1 mm were machined through the brass rods to host the disc magnets. To enable synchronized rotation of the magnets along the fluidic channel, the three brass rods with embedded magnets were assembled in a gearbox and connected to a DC motor (RS336-315, 12 V), which was controlled by a DC power supply (GWINSTEK, GPC-30300). The magnet assembly was hosted in a custom-fabricated acrylic box with a square-shaped opening on the top that enabled the mounting of the microfluidic chip. Each rod carried two sets of magnets, M1 (marked in red) and M2 (marked in blue), which were perpendicular to each other (Figure 1B). The chip was fabricated using a 3D printing technique (Figure 1B, inset), which comprised a long meandering channel and a reservoir (R) at the end of the channel. The chip was mounted above the magnet assembly within a fixed frame that ensured the alignment of the channel to the magnets. When the sample was injected into the channel at an appropriate flow rate (3–5 µL/min), the magnetic beads were subjected to an alternating magnetic field of the magnets. The magnetic field due to the magnets was sufficient to attract the beads within the channel when the magnet’s pole is facing the bottom of the channel, where the distance between the magnet pole and the channel is ~1 mm. The beads aggregated within the magnetic potential well (spot). But, when the magnet rotated 90° such that the pole was perpendicular to the channel, the magnetic field imposed on the beads sharply decreased and became insufficient to hold the beads; therefore, the beads disaggregated and were dispersed within the fluid towards the next potential well to be trapped and aggregate again. Due to the continuous rotation, the trapping and releasing process took place 12 times, corresponding to the number of magnets. Each trapping and releasing event provided a chance to release any impurity from the bead–exosome complex, i.e., a washing cycle took place. The purified bead–exosome sample was finally trapped and collected in a reservoir (R) using a stationary disc magnet (M) at the end of the channel.

### 2.5. RNA Isolation and Quantification

The RNA was isolated from the magnetically pre-concentrated exosomes using an Rneasy RNA isolation kit (Qiagen) according to the manufacturer’s protocol. Briefly, 100 µL of exosomes was disturbed and homogenized in RLT buffer and lysis reagent. Then, 100 µL of chloroform was added, and the solution was mixed and centrifuged. The aqueous layer was collected in a separate tube and 100% ethanol was added. The amount of total RNA extracted from the mixture was quantified by measuring UV absorption at 260 nm.

### 2.6. Reverse Transcription and RT-qPCR

Purified total RNA was used for the production of cDNA from the miRNA using the RT2 first strand kit (Qiagen, cat No 330401) following the manufacturer’s protocol. GC-rich stem-loop primers (Table 2), RT buffer, 0.25 mM dNTPs, and reverse transcriptase were employed for the reverse transcription reaction. A total of 25 µL of the mixtures were incubated in a thermocycler at 16 °C, 42 °C, and 85 °C for 30 min, 30 min, and 5 min, respectively. A total of 2% agarose gel electrophoresis was used to confirm the size of the product obtained from the reverse transcription. Then, the product was purified using a Qiagen DNA purification kit. The amount of cDNA was quantified by measuring UV absorption at 260 nm. The purified cDNA was further aliquoted according to the experimental requirements. Real-time RT-qPCR experiments were carried out in 25 µL using 0.2 µM forward and reverse primers, 200 μM dNTP, and 2 units of Taq polymerase in SYBR green PCR master mix. The PCR mixture was incubated at 94 °C for 5 min, followed by 40 cycles of incubation at 94 °C for 15 s, 55 °C for 30 s, 70 °C for 30 s, and a final extension step of 10 min at 70 °C. The real-time PCR amplification was performed using the fluorescence signal of SYBR green, which acted as a fluorescent probe. The experiments were repeated three times to obtain the concordant values. The threshold cycle (Ct) is the fraction of the cycle at which the fluorescence intensity crosses the threshold for the same cycle number. Finally, from the standard curve, the Ct values were converted into absolute copy numbers.

### 2.7. Magnetic Nanobeads Fluorescence Sensor Platform for Mir210 Detection

The carbon-coated magnetic (CCM) nanobeads play a dual function as a carrier for exosome separation and as a sensing substrate, where the CNM is used as a fluorescence quencher, which forms a major element in the developed sensor. Initially, the fluorescence-based sensing was optimized to ensure that the amount of fluorescently labeled cDNA required for a fixed amount of CNM-coated magnetic beads provided an acceptable signal-to-noise ratio. CCM nanobeads in the dynamic range of 0 to 400 µg/mL were titrated with a fixed amount of FAM-labeled cDNA of microRNA 210. Based on the quenching efficiency of the 35 µg/mL of the CCM nanobeads (25 nM FAM-cDNA to 40 µg/mL nanobeads), the optimized concentration of the FAM-labeled cDNA concentration was fixed at 25 nM. Following optimization, a mixture of 25 nM FAM-cDNA and 35 µg/mL nanobeads was incubated at room temperature for 30 min, which led to significant adsorption of the FAM-cDNA by the CNM. Then, mir210 with a concentration within the dynamic range of 0 to 50 nM with FAM-cDNA/CCM nanobeads in binding buffer (50 mM Tris. HCl + 150 mM NaCl + 2 mM MgCl_2_, pH = 7.4) was incubated for 30–35 min. The fluorescence intensity of each sample was recorded at an excitation/emission of 470 ± 10 nm/515 nm and plotted against the concentration of FAM-cDNA.

### 2.8. Specificity

The assay specificity assay was tested against other closely associated miRNAs, such as mir10b, mir16, and mir191. The miRNA samples were incubated with FAM-labeled mir210 complementary DNA/nanobeads in an optimized ratio. The fluorescence signals were measured and compared for cross-reactivity.

## 3. Results and Discussions

### 3.1. Apta-Magnetic Separation (AMS) and Particle Size Determination of Exosomes

MicroRNAs are stabilized by the biomolecules within the exosomes and bind with proteins in the presence of transmembrane and cytosolic proteins, mRNA, and DNA [24,25,26,27]. In the current separation method (Figure 1A), the sample, with magnetic particle suspension, flows inside a meander-shaped channel that is aligned to the magnet assembly. The rotation of the magnet assembly generates an array of alternating magnetic potential wells due to the two magnet sets (M1 and M2). When the magnetic poles of M1 point directly to the channel, the magnetic beads are exposed to strong magnetic forces; therefore, the beads are trapped and aggregated. Meanwhile, the magnetic forces due to the set M2 have a negligible effect on the beads compared to the inertial force; therefore, they continue to flow downstream and become trapped in the next M1 trapping zone. The alternate rotation of the magnets (i.e., polar orientation) generates a series of alternate “trapping and releasing”, which enables a series of washing events to take place before the final trapping and concentration at the end of the channel due to the stationary magnet M.

### 3.2. Isolation and Measurement of Particle Size of Exosome

**Figure 2:**
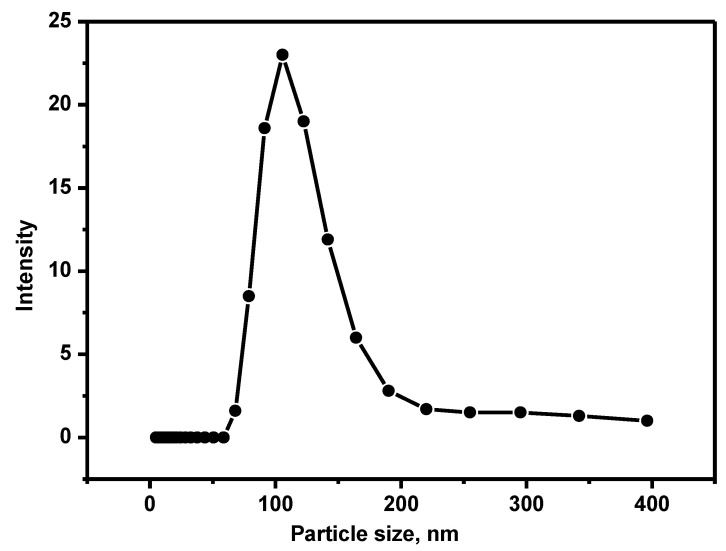
The intensity of the scattered light by the exosome particles isolated from the Luc-4T1 cell, which was excited at a wavelength of 633 nm. The measurements were conducted in triplicate.

The magnetically pre-concentrated exosomes were isolated using the ssDNA complementary sequence of the CEA aptamer. In the presence of the aptamer complementary sequences, the aptamer covalently conjugates to the magnetic beads to form a strong dsDNA duplex. As a result, the aptamer-bound exosomes are released into the solution [28]. Dynamic light scattering with photon correlation spectroscopy was used to measure the exosome size was measured following the method described in [29]. The scattered light from the particles due to the laser beam was correlated with the particle size distribution of the particles (Figure 2). The particle size was estimated to be within the range of 80–120 nm.

### 3.3. CCM Beads as a Fluorescence Sensing Substrate

**Figure 3:**
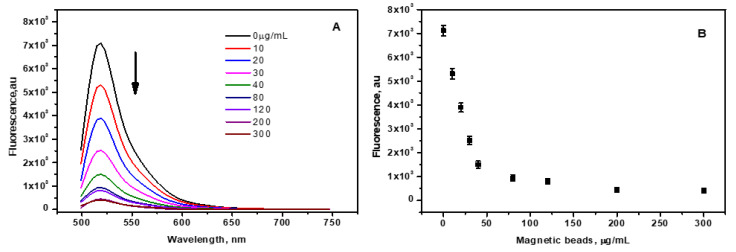
(**A**) The fluorescence intensity of mir210 cDNA decreases with increasing concentrations of the CNM-coated magnetic nanobeads at bead concentrations of 0, 10,20, 30, 40, 80,120, 200, and 300 µg/mL. (**B**) The fluorescence intensity of the FAM-cDNA as a function of the magnetic nanobeads concentration was measured at excitation/emission of 470 ± 10 nm/515 nm. The error bars represent the standard deviation of three different measurements.

Fluorescence switching was used as a non-invasive mechanism to detect the breast cancer biomarker mir210 using CCM nanobeads as a fluorescence quencher and the fluorescent-labelled complementary sequence of mir210 as a sensing probe. It is well known that ssDNA is effectively adsorbed onto the surface of CCMs [30] due to their high surface-to-volume ratio. The CCM nanobeads act as a fluorescence quencher due to the fluorescence resonance energy transfer (FRET) (Figure 1B). CNM can strongly interact with fluorescently labeled ssDNA via non-covalent interactions including, π-stacking, hydrogen bonding, hydrophobic interactions, etc. [31,32]. The fluorescence of FAM-labelled DNA was significantly quenched in the presence of CCM beads (e.g., from 10^3^ au at a CNM concentration of 120 µg/mL to ~7 × 10^3^ au in the absence of CNM) (Figure 3A). However, in the presence of mir210, the complementary sequence is duplexed with the target, and the fluorescence is restored, as illustrated in Figure 1A.

### 3.4. Optimization of CNM/FAM-DNA Ratio (Fluorescence off State)

CCM beads were added to a fixed amount of FAM-DNA, and the change in the fluorescence intensity due to quenching by CNM was monitored. The minimum amount of beads was 25 nM, at which a significant signal-to-noise ratio (s/n) was found. A total of 25 nM of mir210 cDNA was titrated against a variable amount of CCM beads in the dynamic range of 0 to 300 µg/mL. In the absence of magnetic beads, the fluorescence of the FAM-cDNA was stronger (Figure 3A). However, when magnetic beads were added, the fluorescence intensity significantly decreased when the concentration of magnetic beads increased (Figure 3B). The fluorescence intensity reached less than 15% at a magnetic bead concentration of 40 µg/mL. No significant reduction in intensity was observed at higher bead concentrations (e.g., 300 µg/mL compared to 200–300 µg/mL) (Figure 3B). Using this concentration, significant adsorption of mir210 cDNAs by the CCM beads was achieved. When the fluorophore conjugated to the cDNA was excited at 470 ± 10 nm, the emitted photon energy from the fluorophore was transferred to the CNM via the fluorescence resonance energy transfer (FRET) mechanism, which led to a drastic decrease in the signal intensity. Therefore, the optimal bead concentration at which ~80–85% of quenching was obtained was fixed at 40 µg/mL. The optimized ratio of 25 nM of mir210 cDNA for 40 µg/mL was considered as the off state and selected for further experiments.

### 3.5. Mir210 Detection Using CCM Beads Fluorescence Assay

**Figure 4:**
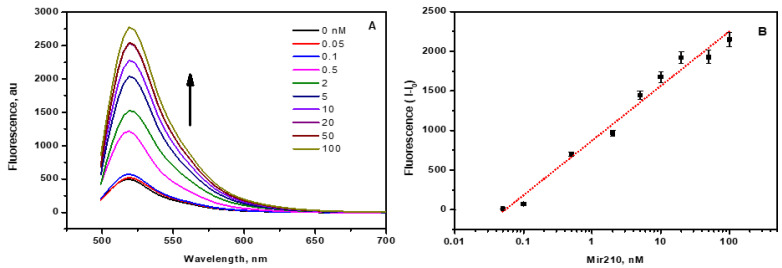
(**A**) FAM-cDNA is released from the magnetic nanobead surface due to the presence of mir210. The fluorescence intensity increased upon increasing the mir210 concentration. The fluorescence intensity was monitored at 515 nm and plotted against the concentration of mir210. (**B**) The standard calibration plot for the mir210 at excitation/emission of 470 ± 10 nm/515 nm. The error bars represent the standard deviation of three different measurements.

When the labelled complementary cDNAs of mir210 came into contact with the CCM beads, they were adsorbed by the CNM via various weak interactions. Hence, a significant quenching of fluorophore-labelled cDNA takes place. Upon the addition of mir210 within the range of 0.05 to 100 nM to the magnetic beads-cDNA complex, the fluorescence intensity increased with increasing concentration of mir210 (Figure 4A). The mir210 formed a double-stranded DNA duplex with FAM-cDNA, which was thermodynamically more stable than the magnetic beads–cDNA complex. Therefore, in the presence of mir210, cDNA detached from the CNM surface and formed mir210-cDNA dsDNA with the maximum number of base pairs in the buffer solution. When FAM-cDNA was detached from the CCM beads, the fluorophore was at a distance from the quenching CNM; thus, the emitting photons from the fluorophore became highly observed, thereby increasing the fluorescence intensity as the mir210 concentration increased. To obtain the calibration curve, the fluorescence intensities due to the presence of mir210 were plotted as a function of the logarithmic concentration of mir210, and a linear relationship was observed (Figure 4B). The limit of detection (LOD) was calculated as 3.3 SD/s, where SD is the standard deviation of the signal of blank samples and S is the slope of the linear calibration curve. The LOD of mir210 was found to be 5 pM. The achieved sensitivity using the current method was significantly higher than that recently reported using mir21cDNA-FAM and graphene oxide, which was 20 pM [33]. The fluorescence intensity of the sample was found to be proportional to the concentration of mir210 present in the sample, which was further confirmed using the standard RT-PCR method in the next sections. Zhou et al. developed a G-quadruplex molecular beacon fluorescence probe for the detection of miRNA using duplex-specific nuclease (DSN) for signal amplification with a detection limit of 1 pM [34]. In another study, a fluorescence switching mechanism was also employed for the detection of miRNA using fluorescent-labelled miRNA-cDNA gold nanoparticles as a fluorescence quencher. The reported LOD of this competitive binding assay was 3.8 pM [35]. A graphene oxide-based fluorescence assay was also developed using duplex-specific nuclease amplification with an LOD of 0.16 pM [36].

### 3.6. Reverse Transcription and RT-qPCR

**Figure 5:**
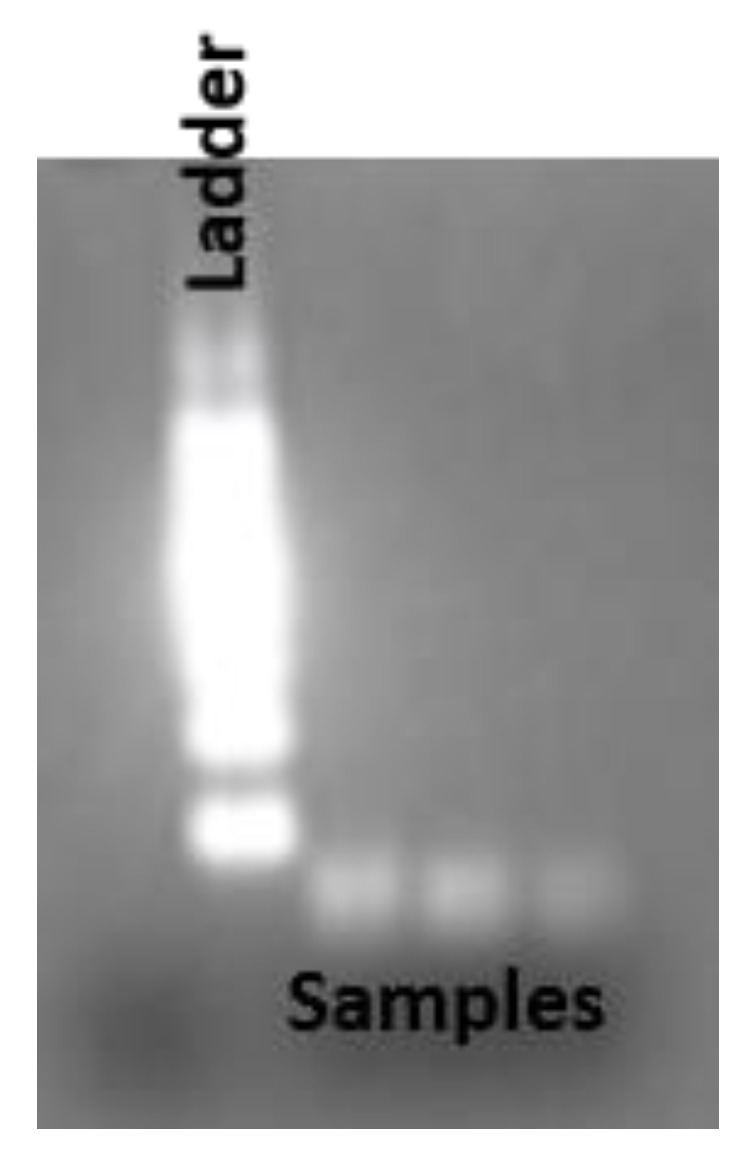
Agarose gel (2%) electrophoresis of reverse transcribed product using total RNA extracted from exosome as a template and the stem-loop primers designed for mir210. The size of the cDNA from the reverse transcription is compared with the 100 bp DNA ladder.

**Figure 6:**
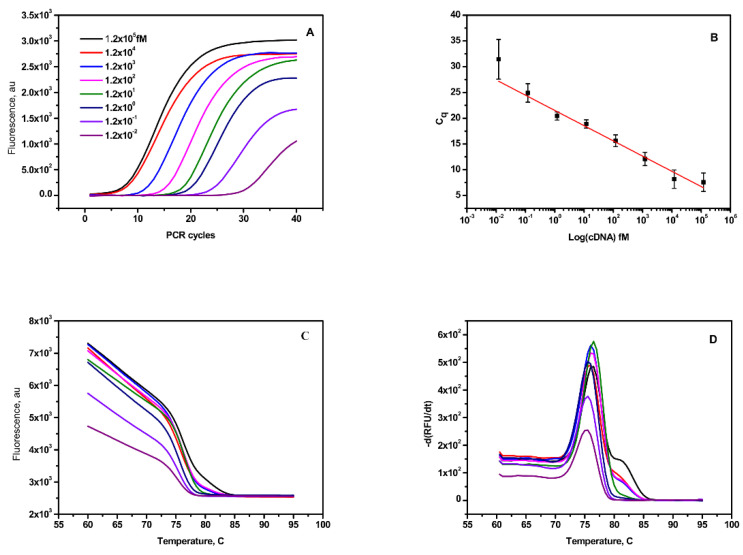
Mir210 RT-qPCR assay. (**A**) PCR amplification plot of mir210 cDNA over seven orders of magnitude from 0.012 fM to 1.2 × 10^5^ fM. (**B**) Standard calibration curve of mir210 cDNA. (**C**) Melting curve of the amplified PCR product in the temperature range between 5 to 95 °C. (**D**) First derivative of the melting curve. The Tm of the products is close to 75.5 ± 1.5 °C.

The current fluorescence switching assay was compared with the RT-qPCR method, which is a standard method for the quantification of miRNA. The RT-qPCR process requires two steps, namely reverse transcription and amplification. The RT primer with a stem-loop structure hybridizes with mi210 and produces complementary DNA of mir210 cDNA by reverse transcription. The desired size of the reverse-transcribed cDNA was confirmed using 2% agarose gel electrophoresis (Figure 5) and purified using a PCR purification kit. The quantity of cDNA was calculated to be 12 ng/µL based on the UV absorption at 260 nm. The stock cDNA was aliquoted further to obtain a variable concentration range of the template for RT-qPCR using SYBR green as the fluorescent probe. cDNA samples with higher concentrations reached the threshold value of the fluorescence intensity with a smaller number of amplification cycles, whereas more cycles of amplification are needed in the case of diluted cDNA samples (Figure 6A), which implies that the PCR mixture with more concentrated cDNA template has a lower threshold cycle (Ct) and vice versa. The plot of. Ct values against the log value of cDNA input in the variable concentration range of 1.2 × 10^−2^ fM 1.2 × 10^5^ fM to (35 copies to 108 copies of the template) showed perfect linearity as depicted in Figure 6B. No change in the fluorescence of SYBR green was observed during the thermal cycles for the negative control (without mir210 or other DNA templates). The achieved sensitivity enabled the detection of as low as 100 copies in the sample. The lowest detectable copies of mir210 cDNA were obtained using the formula 3 × STD/q, where STD is the standard deviation of the RUF value in the absence of a cDNA template, and q is the slope of the linear fit. To confirm that the desired product was obtained from the PCR amplification processes, the melting curve of each sample was observed from the fluorescence of the SYBR green to avoid the non-specific amplification products such as primer-dimer (Figure 6C). The sharp peaks at 75.5 ± 1.5 °C (Figure 6D) in the second derivatives of the melting curves confirmed that the amplified PCR products had melting temperatures in the range between 75 °C and 80 °C. The lowest detection amount of RT-qPCR was relatively very low in comparison with the fluorescence switching assay; however, unlike PCR, the fluorescence switching assay is a direct measurement without any further enzymatic amplification. As RT-qPCR technique is expensive and needs highly sophisticated instruments, expert technicians, suitable primer setup for the targets, and storage and stability issues, which make it unsuitable for point-of-care applications.

### 3.7. Cross-Reactivity and Specificity

**Figure 7:**
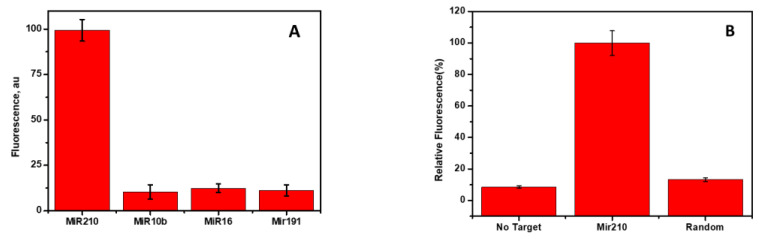
(**A**) The fluorescence signal due to the addition of different miRNAs to the cDNA adsorbed on the CNM surface. The concentrations of mir210, mir10b, mir16, and mir191 are 50 nM in the binding buffer. (**B**) Change in the fluorescence signal in the presence of mir210, without target and non-specific scrambled DNA. The fluorescence signals were recorded at excitation/emission of 470 ± 10 nm/515 nm. The error bars represent the standard deviation of three different measurements.

The specific binding of mir210 with the labelled cDNA, which was adsorbed onto the CCM beads, was tested using the closely related miRNA sequences. The target, mir210, and other nonspecific miRNAs such as mir10b, mir16, and mir191 were incubated and the changes in the fluorescence intensity were monitored. As shown in Figure 7A, mir210 showed the maximum fluorescence signal. However, there was no significant change in the signal when other target sequences (mir16, mir191, and mir10b) were used compared to the mir210 miRNA target. Similar results were observed without target mir210 and/or using scrambled DNA (Figure 7B), which confirms the high selectivity of the current method for the target miRNA.

The method developed in the current study is simple and provides a fast assay (~30 min) without the need for signal amplification compared to the above-mentioned methods. The high signal-to-noise ratio is multiplied by the amplification or the reproduction of the probing DNA by enzymes. The current method is compactable for the in vivo diagnosis application with a slight chemical modification in the DNA backbone or 2’OMe modified RNA [37], or by blocking the 3’end of the cDNA to protect from the nuclease digestion. 

## 4. Conclusions

A CNM-based fluorescence switching assay was developed for the detection of mi210 biomarkers using FAM-labeled cDNAs. The aptamer-based microfluidic separation and purification of exosomes were applied and particle size was determined. The exosomal mir210 was extracted and quantitatively detected by the fluorescence switching method using CCM beads as a fluorescence quenching platform. The limit of detection of the method was calculated to be 5 pM using the standard calibration curve. No significant cross-reactivity with the other closely related miRNAs was observed. The fluorescence method was validated using the standard RT-qPCR amplification techniques and miRNA biomarker screening via high-throughput methods from the exosomes for specific disease diagnosis, prognosis, and point-of-care application. This method is simple and can be used for the detection of miRNAs at the point of care. The device can be further optimized to manipulate smaller sample volumes and can be used for the detection of other analytes from different types of samples.

## Figures and Tables

**Table 1:** Synthetic Mir210 and other oligonucleotides used in this work.

Aptamer	Oligonucleotides (5′-3′) Used
Mir210	CUGUGCGUGUGACAGCGGCUGA
Mir210 cDNA	GACACGCACACTGTCGCCGACT
Reverse complement	FAM-TCAGCCGCTGTCACACGCACAG
Anti-CEA Aptamer	H_2_NTCGCGCGAGTCGTCTGGGGAACCATCGAGTTACACCGACCTTCTATGTGCGGCCCCCCGCATCGTCCTCCC
Reverse complementary of CEA aptamer	GGGAGGACGATGCGGGGGGCCGCACATAGAAGGTCGGTGTAACTCGATGGTTCCCCAGACGACTCGCGCGA

**Table 2:** Reverse transcription primers and final product.

	Primers
Reverse transcription primer	GTCGTATCCAGTGCAGGGTCCGAGGTATTCGCACTGGATACGACTCAGCC
Forward primer	GTATACCTGTGCGTGTGACAG
Reverse primer	GTGCAGGGTCCGAGGT
Final product	5′-GTGCAGGGTCCGAGGTATTCGCACTGGATACGACTCAGCCGCTGTCACACGCACAGGTATAC-3′ 3′-CACGTCCCAGGCTCCATAAGCGTGACCTATGCTGAGTCGGCGACAGTGTGCGTGTCCATATG-5′

## Data Availability

The data presented in this study are available on request from the corresponding author.
